# Olanzapine: A potent agonist at the hM4D(Gi) DREADD amenable to clinical translation of chemogenetics

**DOI:** 10.1126/sciadv.aaw1567

**Published:** 2019-04-17

**Authors:** Mikail Weston, Teresa Kaserer, Angela Wu, Alexandre Mouravlev, Jenna C. Carpenter, Albert Snowball, Samuel Knauss, Melanie von Schimmelmann, Matthew J. During, Gabriele Lignani, Stephanie Schorge, Deborah Young, Dimitri M. Kullmann, Andreas Lieb

**Affiliations:** 1 Department of Clinical and Experimental Epilepsy, UCL Queen Square Institute of Neurology, University College London, London, UK.; 2 Cancer Research UK Cancer Therapeutics Unit, The Institute of Cancer Research, London SM2 5NG, UK.; 3 Department of Pharmacology & Clinical Pharmacology, The University of Auckland, Auckland, New Zealand.; 4 Ovid Therapeutics, Inc., 1460 Broadway, New York, NY 10036, USA.

## Abstract

Designer receptors exclusively activated by designer drugs (DREADDs) derived from muscarinic receptors not only are a powerful tool to test causality in basic neuroscience but also are potentially amenable to clinical translation. A major obstacle, however, is that the widely used agonist clozapine *N*-oxide undergoes conversion to clozapine, which penetrates the blood-brain barrier but has an unfavorable side effect profile. Perlapine has been reported to activate DREADDs at nanomolar concentrations but is not approved for use in humans by the Food and Drug Administration or the European Medicines Agency, limiting its translational potential. Here, we report that the atypical antipsychotic drug olanzapine, widely available in various formulations, is a potent agonist of the human M4 muscarinic receptor-based DREADD, facilitating clinical translation of chemogenetics to treat central nervous system diseases.

## Teaser

The atypical antipsychotic olanzapine is a potent activator of a mutated muscarinic receptor used for chemogenetic inhibition.

## INTRODUCTION

Central nervous system (CNS) diseases caused by abnormal circuit function represent a major burden to society. Although many respond to conventional small-molecule treatment, some diseases such as intractable pain and refractory epilepsy account for a substantial unmet need. Drug-resistant focal epilepsy alone affects approximately 0.2% of the entire population (*1*, *2*). Although surgical resection of the epileptogenic zone is effective, it is contraindicated in the overwhelming majority of patients because of high risks of permanent disability associated with brain tissue removal (*3*). Several gene therapies for refractory epilepsy, based on altering the balance of excitation and inhibition, have been validated in preclinical models (*4*–*9*). Chemogenetics using viral vector–mediated expression of the inhibitory muscarinic M4 receptor–based Gi-coupled DREADD (designer receptor exclusively activated by designer drug) hM4D(Gi) is especially promising because the therapeutic effect can be titrated by adjusting the dose of the activating ligand (*10*). Several recent publications have shown that hM4D(Gi) expressed in epileptogenic zones can suppress partial-onset seizures when activated (*5*, *11*, *12*).

A potential limitation to clinical translation of DREADD technology is that most studies to date have used clozapine *N-*oxide (CNO), the inactive metabolite of the atypical antipsychotic drug clozapine (CZP) (*13*), as the ligand. CNO is not approved for clinical use, and recent evidence shows that CNO is actively exported from the CNS and back-converted to CZP, which crosses the blood-brain barrier and subsequently acts as the ligand activating the DREADD (*14*, *15*). CZP as an activator of hM4D(Gi), however, represents major logistical and regulatory obstacles because it has an unfavorable side effect profile, including a risk of agranulocytosis and myocarditis, and can reduce seizure threshold (*16*–*18*). Related antipsychotic drugs have been proposed as potential agonists (*13*, *14*), and two other drugs activating DREADDs have recently been described: “compound 21” (C21) and perlapine (PLP) (*19*, *20*). Although PLP has previously been used as a mild sedative antihistamine drug in Japan, neither it nor C21 is approved for clinical use by the Food and Drug Administration (FDA) or the European Medicines Agency (EMA). Identification of an FDA/EMA-approved drug for repurposing as a DREADD activator would facilitate clinical translation of DREADD technology to treat CNS diseases.

## RESULTS

### hM4D(Gi)-dependent Kir3.1 and Kir3.2 activation

**Fig. 1:**
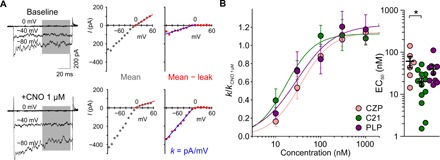
Electrophysiology-based screen of hM4D(Gi) activation. (**A**) Left: Representative traces of Kir3.1 and Kir3.2 currents with (+CNO 1 μM, bottom) and without (baseline, top) hM4D(Gi) agonist application. Middle: Mean current measured during the time indicated by the gray area in the left panel, plotted against holding voltage. The red line indicates the calculation of the membrane leak conductance, obtained from a linear fit between 0 and +50 mV. Right: Leak-subtracted Kir3.1/Kir3.2-mediated currents, together with a linear fit to currents at negative potentials (blue). The slope of the current-voltage relationship (*k*) was used for subsequent analysis of hM4D(Gi) activation. (**B**) Left: CZP, C21, and PLP act as potent agonists of hM4D(Gi). All data are shown normalized to CNO (1 μM) as a positive control and fitted by a Hill equation. Right: EC_50_ of CZP, C21, and PLP (CZP: EC_50_ = 61 ± 19 nM, *n* = 6; PLP: EC_50_ = 40 ± 10 nM, *n* = 9; C21: EC_50_ = 20 ± 4 nM; *P < 0.05, one-way ANOVA with Bonferroni post hoc test).

**Fig. 2:**
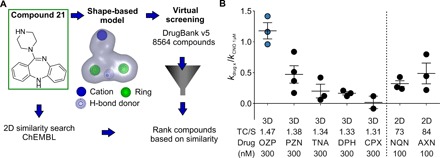
Shape- and 2D similarity–based identification of hM4D(Gi) agonists. (**A**) Overview of the 3D- and 2D-based virtual screen. C21 was used as the query compound for both the 2D similarity search of the ChEMBL database and the generation of a 3D shape–based model, because it showed the lowest EC_50_ at hM4D(Gi) of known agonists. For detailed virtual screening results, see table S1. (**B**) hM4D(Gi)-dependent potentiation of Kir3.1/Kir3.2-mediated currents measured for selected hit compounds is normalized by 1 μM CNO as positive control. The TanimotoCombo (TC) score (for 3D screen), similarity (S) index (for 2D screen), the screening method (3D or 2D; separated by dotted line), and the tested concentration are indicated. For detailed structures of selected drugs, see table S2.

To measure Gi-coupled hM4D(Gi) activation, we established an electrophysiological screen based on measuring the potentiation of the inward-rectifying potassium current in a human embryonic kidney cell line stably expressing Kir3.1 and Kir3.2 (Fig. 1A) (*21*). We verified the sensitivity of the system by estimating the half-maximal effective concentration (EC_50_) of CZP as 61 ± 19 nM (mean ± SEM; Hill coefficient, 1.44 ± 0.28; *n* = 6), close to the reported EC_50_ of 57 nM (*13*). We used CNO (1 μM) as a positive control to define maximal activation of hM4D(Gi) (Fig. 1B) and confirmed that CZP, PLP, and C21 are efficacious agonists (maximal activation of inward-rectifying current in comparison to 1 μM CNO: CZP/CNO = 1.14 ± 0.06, *n* = 6; PLP/CNO = 1.17 ± 0.16, *n* = 9; C21/CNO = 1.11 ± 0.07). C21 showed a significantly lower EC_50_ than CZP [CZP EC_50_ = 61 ± 19 nM; PLP EC_50_ = 40 ± 10 nM; Hill coefficient, 1.69 ± 0.39; C21 EC_50_ = 20 ± 4 nM; Hill coefficient, 1.36 ± 0.22; EC_50_ difference *P* < 0.05, one-way analysis of variance (ANOVA) with Bonferroni post hoc test]. Although C21 is a potent agonist of hM4D(Gi), additional pharmacokinetic and safety characterization would be required before clinical translation (*19*). We therefore performed a shape [three-dimensional (3D)] (*22*, *23*) and 2D similarity screen (*24*) to identify FDA/EMA-approved drugs with structural and electrochemical properties similar to those of C21 (Fig. 2A). Prioritized drugs with similarity indicated by the TanimotoCombo score for the 3D screen, and by similarity for the 2D-based screen, are listed in Fig. 2B and table S1.

### hM4D(Gi) activation with drugs identified by similarity screens

**Fig. 3:**
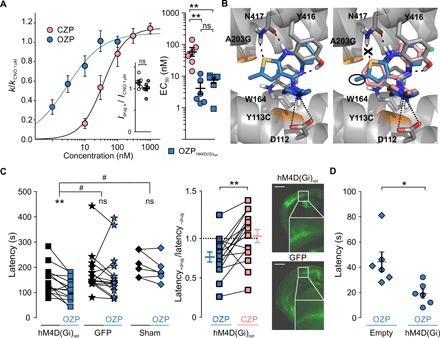
OZP is a potent agonist at hM4D(Gi). (**A**) Left: Dose-response curves for CZP and OZP at hM4D(Gi). The inset shows the efficacy of OZP (100 nM) and CZP (1 μM), normalized to 1 μM CNO as a positive control (CZP/CNO = 1.14 ± 0.06; *n* = 6; OZP/CNO = 1.01 ± 0.06; *n* = 6; *P* = 0.12, Student’s *t* test). Right: EC_50_ for CZP at hM4D(Gi), and OZP at hM4D(Gi) and at the codon-optimized hM4D(Gi)_opt_ [CZP: hM4D(Gi) EC_50_ = 61 ± 19 nM, *n* = 6; OZP: hM4D(Gi) EC_50_ = 5 ± 2 nM, *n* = 6, *P* < 0.01 in comparison to CZP hM4D(Gi); OZP: hM4D(Gi)_opt_ EC_50_ = 7 ± 2 nM, *n* = 6, *P* < 0.01 in comparison to CZP hM4D(Gi); one-way ANOVA with Bonferroni post hoc test]. ns, not significant. (**B**) Docking poses of OZP (left, blue sticks) in comparison to CZP (right, rose sticks) in the homology model of active hM4D(Gi) (gray cartoon and sticks). Ionic interactions with D112 and hydrogen bonds are highlighted by dotted and dashed lines, respectively. The methyl group of OZP is highlighted in the right panel. The Y113C and A203G mutations are highlighted in orange, and residues 434 to 443 are not depicted for clarity. (**C**) Left: Latency to fall of animals injected with AAV2/8-hCamKII-hM4D(Gi)_opt_ [before OZP: 149 ± 14 s; after OZP (0.1 mg/kg, i.p.): 111 ± 11 s; *n* = 15; **P = 0.002, paired Student’s *t* test], those injected with AAV2/8-hCamKII-GFP (before OZP: 180 ± 21 s; after OZP: 169 ± 25 s; *n* = 15), and sham-injected animals (before OZP: 212 ± 21 s; after OZP: 189 ± 20s; *n* = 6; **P < 0.01, Student’s paired *t* test; ^#^*P* < 0.05, repeated-measures ANOVA with LSD post hoc test). Middle: Normalized latency to fall of AAV2/8-hCamKII-hM4D(Gi)_opt_–injected animals treated with either OZP (0.1 mg/kg) (0.76 ± 0.06; *n* = 15) or CZP (0.1 mg/kg) (1.03 ± 0.08; *n* = 15; **P = 0.033, Student’s paired *t* test). Right: Representative confocal fluorescence images of mouse brains injected with either AAV2/8-hCamKII-hM4D(Gi)_opt_ (DREADD, −1.3 mm from bregma) or AAV2/8-hCamKII-GFP (GFP, −1.2 mm from bregma) (scale bars, 1 mm). (**D**) Latency to fall after OZP (0.1 mg/kg, i.p.) in animals injected with either AAV2/5-hCamKII-hM4D(Gi) or AAV2/5-hCamKII-empty bilaterally into striatum [AAV2/5-hCamKII-hM4D(Gi), 19 ± 4 s; *n* = 6; AAV2/5-hCamKII-empty, 44 ± 8 s; *n* = 6; *P* = 0.014, Student’s unpaired *t* test].

We tested olanzapine (OZP; 3D rank 1), promazine (PZN; 3D rank 2), tripelennamine (TNA; 3D rank 5), diphenhydramine (DPH; 3D rank 6), chlorprothixen (CPX; 3D rank 9), and amoxapine (AXN; 2D rank 2). We also tested the first, putative active metabolite of quetiapine (2D rank 6), norquetiapine (NQN) (*25*) (for chemical structures of all tested molecules, see table S2). Of all the drugs tested, only OZP was able to fully activate hM4D(Gi) at a concentration between 100 and 300 nM using 1 μM CNO as control as above (OZP/CNO = 1.18 ± 0.13; *n* = 3) (Fig. 2B). A full dose-response curve for OZP revealed an EC_50_ of 5 ± 2 nM (Hill coefficient, 1.11 ± 0.25; *n* = 6), significantly lower than that of CZP (EC_50_ = 61 ± 19 nM, *n* = 6; *P* = 0.0128, Student’s *t* test) (Fig. 3A). To gain further insights into the observed activity differences on a molecular level, we docked OZP, CZP, and CPX into an active-state homology model of hM4D(Gi) using an induced fit procedure. In addition to the ionic interactions with D112, the docking poses of OZP suggest stacking interactions with W164 and hydrogen bonds involving Y116 and N117 (Fig. 3B). In addition, the OZP methyl group extends into a side pocket that is also occupied by the agonist iperoxo in the hM2 crystal structure complex [Protein Data Bank (PDB) entry 4MQS (*26*); fig. S1A]. This hydrophobic pocket is less occupied by CZP, which, in combination with lack of a hydrogen bond with the N117 amino group (Fig. 3B), could explain the lower activity of CZP compared to OZP. In contrast, the geometry of the inactive CPX and its lack of heteroatoms prevent the formation of any of the hydrogen bonds observed for OZP and position the basic moiety further away from D112 (fig. S1B).

### In vivo verification that OZP activates hM4D(Gi)

To test whether OZP is effective in vivo, we redesigned a codon-optimized version of hM4D(Gi) linked via a viral self-cleaving 2A peptide to green fluorescent protein (GFP) [hM4D(Gi)_opt_] and put it under control of a human Ca^2+^/calmodulin-dependent protein kinase IIα (CamKIIα) promoter (hCamKII) for preferential expression in forebrain principal neurons (*27*). We verified that the EC_50_ of OZP at hM4D(Gi)_opt_ (7 ± 2 nM; Hill coefficient, 1.20 ± 0.17; *n* = 6) was similar to the EC_50_ at the original hM4D(Gi) (5 ± 2 nM, *n* = 6) (Fig. 3A, right). Postnatal day 0 (P0) mice were randomized for injection of either 2.5 μl of AAV (adeno-associated virus) 2/8-hCamKII-hM4D(Gi)_opt_ or 2.5 μl of AAV2/8-hCamKII-GFP (control) into both lateral ventricles (Fig. 3C, right). A third group of mice received no injection. After a period of training, their performance on the rotarod was then tested at ~P42, while blinded to the viral injection. All mice received an acclimatization session on the rotarod on the day of testing, followed by two test sessions, one before and one after ligand injection. We injected OZP at a dose of 0.1 mg/kg intraperitoneally (i.p.). This dose corresponds to a peak plasma concentration below that which is associated with weight gain in chronic treatment, one of the most commonly reported side effects of OZP therapy (*28*, *29*). OZP significantly reduced the latency to fall from 149 ± 14 s to 111 ± 11 s (*n* = 15; *P* = 0.002, Student’s paired *t* test) in AAV2/8-hCamKII-hM4D(Gi)_opt_–injected animals. OZP had no effect on AAV2/8-hCamKII-GFP–injected animals [control, 180 ± 21 s; OZP, 169 ± 25 s; *n* = 15; *P* = 0.568, Student’s paired *t* test; comparison with AAV2/8-hCamKII-hM4D(Gi)_opt_, *P* = 0.045, repeated-measures ANOVA with LSD post hoc test] or on mice that had received no viral injection [control, 212 ± 21 s; OZP, 189 ± 20s; *n* = 6; *P* = 0.109, Student’s paired *t* test; *P* = 0.028, repeated-measures ANOVA with least significant difference (LSD) post hoc test] (Fig. 3C, left). A similar dose of CZP (0.1 mg/kg) had no effect on the same AAV2/8-hCamKII-hM4D(Gi)_opt_–injected animals, where OZP was effective [control, 150 ± 18 s; CZP (0.1 mg/kg), 143 ± 12 s; *P* = 0.593, Student’s paired *t* test]. Expressing the effect of hM4D(Gi) activation as a ratio of latency to fall with and without drug (latency_+drug_/latency_–drug_), AAV2/8-hCamKII-hM4D(Gi)_opt_–injected animals were significantly more sensitive to OZP (0.76 ± 0.06; *n* = 15) than CZP (1.03 ± 0.08; *n* = 15; *P* = 0.003, Student’s paired *t* test) (Fig. 3C, middle). The failure of CZP (0.1 mg/kg) to alter motor behavior contrasts with a previous report (*15*), consistent with a relatively low level of hM4D(Gi) expression in the present study.

Last, we removed the fluorescent tag from the original hM4D(Gi) and injected either AAV2/5-hCamKII-hM4D(Gi) or a control empty vector (AAV2/5-hCamKII-empty) bilaterally into the striatum of adult rats. After four training sessions to acclimatize animals, during which the two groups of rats performed equivalently, we tested the effect of OZP (0.1 mg/kg). The latency to fall in animals injected with AAV2/5-hCamKII-hM4D(Gi) (19 ± 4 s, *n* = 6) was significantly shorter than that in animals injected with empty vector control (AAV2/5-hCamKII-empty) (44 ± 8 s, *n* = 6; *P* = 0.014, unpaired Student’s *t* test) (Fig. 3D).

## DISCUSSION

Although recent papers highlight the potential of PLP or C21 as potent activators of hM4D(Gi) (*19*), these ligands would require extensive screening to be approved for clinical use (*30*). PLP was marketed in Japan but was subsequently withdrawn, calling for an alternative licensed drug that can be repurposed as an activator of hM4D(Gi) for clinical translation of DREADD technology. The present study shows that OZP (ranked first in the 3D-based in silico screen) is a potent activator of hM4D(Gi). OZP is a second-generation atypical antipsychotic, which is approved by the FDA and EMA for treatment of schizophrenia and manic episodes in bipolar disorder. Common side effects of OZP at doses used in schizophrenia and bipolar disorder include weight gain, postural hypotension, and sedation (*31*). OZP is a D2 receptor antagonist, and its side effect profile therefore also includes akathisia, tardive dyskinesia, and neuroleptic malignant syndrome, although these are much less common than for first-generation antipsychotic drugs such as haloperidol and chlorpromazine. The in vitro EC_50_ of OZP at hM4D(Gi) is in the range of affinities reported for its native drug targets (table S3) (*32*). The ability to affect performance on the rotarod with OZP (0.1 mg/kg) reported here is consistent with the principle of receptor reserve, whereby GPCR (heterotrimeric guanine nucleotide–binding protein–coupled receptor)–mediated effects can be achieved with low doses of agonist (*33*). Given that CZP is typically only prescribed for treatment-resistant patients because of its unfavorable side effect profile (*34*), CZP is much less suitable for repurposing as a DREADD activator. Nevertheless, the side effect profile of each activator must be considered and determined individually for every potential clinical application of hM4D(Gi). We therefore propose that OZP, which is widely available in oral, intramuscular, and intravenous formulations, is suited for clinical translation of hM4D(Gi)-based chemogenetics to treat CNS diseases, including refractory epilepsy.

## MATERIALS AND METHODS

### Voltage clamp recordings

Kir3.1/3.2 stable expressing cell line (*35*) was cultured in Dulbecco’s modified Eagle’s medium GlutaMax (Gibco), supplemented with 10% fetal bovine serum (Gibco) and penicillin/streptomycin (50 IU/ml; Gibco), and contained Geneticin (500 μg/ml) (Gibco) as a selection marker. Cells were transiently transfected with TurboFect transfection reagent (Thermo Fisher Scientific) with 3 μg of hM4D(Gi) plasmid (Addgene; 45548) and 1 μg of cytomegalovirus promoter (CMV)–GFP for cell identification. Standard whole-cell patch-clamp experiments were performed after 2 to 3 days as previously described (*27*). Briefly, borosilicate-glass electrodes were pulled (Sutter Instrument) and fire-polished (Narishige) with a final resistance of 2 to 4.5 megohms. The extracellular recording solution contained 140 mM KCl, 2.6 mM CaCl_2_, 1.2 mM MgCl_2_, and 10 mM Hepes, adjusted to pH 7.4 with KOH. The intracellular recording solution contained 107 mM KCl, 1.2 mM MgCl_2_, 1 mM CaCl_2_, 10 mM EGTA, 5 mM Hepes, 2 mM Mg-ATP, and 0.3 mM NA_2_-GTP, adjusted to pH 7.2 with KOH. Cells were voltage-clamped at a holding potential of 0 mV, and a 100-ms step depolarization from –100 to +50 mV was applied in 10-mV increments and a 30-s interpulse interval. Whole-cell currents were low-pass–filtered at 2 kHz (Axopatch-1D; Axon Instruments) and digitized at 10 kHz. The membrane leak conductance in each cell was estimated from a linear fit to currents measured between 0 and +50 mV. The inward-rectifying conductance mediated by Kir3.1/3.2 was estimated from a linear fit to currents between −100 and 0 mV after subtracting the leak conductance (Fig. 1A). All recordings were performed at room temperature, and the different drugs were applied by a custom-built perfusion system. CNO (Generon; #HY17366), PLP (Tocris Bioscience; #5549), C21 (Hello Bio; #HB6124), CZP (Cayman Chemical; #12059), OZP (Santa Cruz Biotechnology; #sc-212469), PZN (Sigma-Aldrich; #46674), TNA (Santa Cruz Biotechnology; #sc-229608), DPH (Cerilliant; #D-015), CPX (Santa Cruz Biotechnology; #sc-211077), NQN (BioVision; #2362), and AXN (LKT Laboratories; #A5059) were dissolved in either dimethyl sulfoxide (DMSO) or extracellular recording solution at a stock concentration of 1 mM and subsequently diluted to specified concentrations. CNO (1 μM) was routinely tested to estimate maximal activation of hM4D(Gi) in each cell, and the Kir3.1/3.2-mediated conductance activated by each agonist application was therefore related to that evoked by 1 μM CNO.

### Molecular biology

The hM4D(Gi) plasmid was purchased from Addgene (#45548). Standard molecular biology techniques were used to clone GFP-T2A into an AAV2 transfer plasmid (GeneOptimizer, GeneArt; Thermo Fisher Scientific). The codon-optimized version of HA-hM4D(Gi) full sequence is available upon request) was linked to GFP via a viral 2A peptide, and contained a woodchuck hepatitis posttranscriptional regulatory element (WPRE). For all in vivo experiments, the CMV promoter was replaced with a 1.3 kb CamKIIα promoter to allow expression in excitatory neurons (*27*), the antibiotic resistance was changed from ampicillin to kanamycin, and a restriction site after the 2A peptide was removed. A stop codon and restriction site after the hM4D(Gi) reading frame was inserted using polymerase chain reaction methods to facilitate excision of a nontagged hM4D(Gi) from an hM4D(Gi) mCherry plasmid (Addgene; #50477). The untagged hM4D(Gi) fragment was cloned into an AAV expression plasmid under the control of the human CamKIIα promoter and containing a WPRE and bovine growth hormone polyA and flanked by AAV2-inverted terminal repeats. AAV8 or AAV5 serotype vectors were packaged using methods described previously (*36*).

### In silico screening

One low-energy conformation of C21 calculated with Omega 2.3.2 (*37*, *38*) was used as the query for the generation of the shape-based model. The default model was modified, and the final model only contained the color features shown in Fig. 2A. A maximum number of 200 conformers were generated for DrugBank version 5.0.7 (*39*) with Omega 2.3.2 (*37*, *38*). The default settings of vROCS 3.0.0 (*22*, *23*) were used for screening, and hits were ranked according to the TanimotoCombo score. The ChEMBL (*24*) web service (www.ebi.ac.uk/chembl/; access date 21 June 2017) was used to find FDA/EMA-approved drugs with similar 2D structure to C21.

### Homology modeling and docking

The crystal structure of hM2 in the active state in complex with the agonist iperoxo [PDB entry 4MQS (*26*)] was used as a template to create a homology model of active hM4 in MOE 2018.0101 (*40*). The hM4 sequence used for homology modeling already contained the Y113C and A203G mutations. The default settings were applied, except that iperoxo was considered during model generation and refinement. Iperoxo from the hM2 crystal structure was copied into the hM4 model, and the complex was prepared using the Protein Preparation Wizard (*41*, *42*) in Maestro release 2017-2 (*43*). The ligands were docked into the hM4 model using induced fit docking in Maestro release 2017-2. Redocking was performed with XP settings; otherwise, the default parameters were applied. Figures of the structures and docking poses were created with PyMOL (*44*).

### Viral injections

All animal procedures were performed in accordance with the University College London and the University of Auckland animal care committee’s regulations. Viral aliquots of AAV2/8-CamKIIα-GFP-T2A-hM4D(Gi)_opt_ or AAV2/8-CamKIIα-GFP (both titers, >10^11^ GC/ml; VectorBuilder) were prepared and coded by a researcher conducting neither surgical procedures nor behavioral analyses. P0 neonatal CL57BL/6 mice were anesthetized with intraperitoneal ketamine (6 mg/kg) and midazolam (0.2 mg/kg). A 10-μl microinjection syringe fitted with a 32-gauge angled needle (Hamilton) was filled with one virus. Mouse pups (*n* = 30) were divided equally between viral types and manually injected with 2.5 μl into each lateral ventricle, approximately 1 mm lateral from the sagittal suture and halfway between lambda and bregma, to optimize widespread cerebral transduction. Six pups received no injection. Pups’ paws were marked with green tattoo ink to allow differentiation between viral types, and after recovery, they were returned to their home cage. Male adult Sprague-Dawley rats (150 to 200 g) received a 3-μl injection (200 nl/min) of AAV2/5-hCAMKII-hM4D(Gi) (4.36 × 10^12^ GC) or AAV2/5-hCamKII empty vector (4.14 × 10^12^ GC) bilaterally into the striatum (coordinates from bregma: anterior-posterior, 1.0 mm; medial-lateral, ±2.6 mm; dorsal-ventral, –5.5 mm), with a 33-gauge Neuros syringe (Hamilton).

### Behavioral analysis

At P35, mice were trained on a mouse rotarod (Ugo Basile). Mice were initially acclimatized for 10 min on the rotarod turning at 5 rpm, replacing them each time they fell off, followed by acceleration over a 5-min period from 5 to 40 rpm. The sequence was repeated four times. The latency to fall or to three consecutive cartwheels was recorded. Training was repeated daily until every mouse’s performance reached a plateau, taking approximately 2 weeks.

On the day of DREADD agonist testing, the mice had a further acclimatization session (5 min at 5 rpm, followed by four accelerations), followed by a break of at least 30 min. They were then tested twice, with the same protocol as the acclimatization session, before, and 20 min after intraperitoneal injection of OZP (0.1 mg/kg). OZP and CZP were dissolved in 0.5% DMSO/0.9% NaCl to a concentration of 0.01 mg/kg before injection. The latency to falling off or cartwheeling was recorded.

To test rotarod performance in rats, animals were initially trained 4 weeks after virus injection, on four consecutive days at a fixed rotation speed of 10 rpm (three trials on each day), and allowed to remain on the rotarod for up to 300 s. All six control rats and five of six rats in the AAV2/5-hCamKII-hM4D(Gi) group learned to remain on the rotarod for 300 s in the final training session. On day 5, rats were administered OZP (0.1 mg/kg, i.p.) and tested on an accelerating rotarod 10 min later (with a start speed of 4 rpm, reaching a final speed of 20 rpm after 30 s). Behavioral tests were performed by a researcher blinded to viral treatment.

### Confocal fluorescence

To establish the extent of viral transduction, mice were anesthetized with pentobarbital (150 mg/kg) (Boehringer Ingelheim) and transcardially perfused with 20 ml of heparinized (80 mg/liter) phosphate-buffered saline (Sigma-Aldrich) until the perfusate was clear and then with 4% paraformaldehyde (PFA; Tocris Bioscience) (20 ml). Brains were extracted and immersed in 4% PFA for a further 24 hours before coronal vibratome slicing (Leica VT1000 S) at 50 μm, mounting on slides with VECTASHIELD mounting medium with 4′,6-diamidino-2-phenylindole (Vector Labs), and confocal fluorescence imaging (Zeiss LSM 710) to visualize GFP expression.

### Statistical analysis

Statistical analysis was performed with GraphPad Prism 5.01 or IBM SPSS 22.0.0.0. Student’s unpaired/paired *t* test, one-way ANOVA with Bonferroni post hoc test, or repeated-measures ANOVA with LSD post hoc test was used as indicated. Data are shown as mean ± SEM, and the significance level was set to an α of 0.05.

## Supplementary Material

http://advances.sciencemag.org/cgi/content/full/5/4/eaaw1567/DC1

Download PDF

## SUPPLEMENTARY MATERIALS

Supplementary material for this article is available at http://advances.sciencemag.org/cgi/content/full/5/4/eaaw1567/DC1 Fig. S1. Docking poses of OZP and CPX. Table S1. Hit list of the 3D- and 2D-based screens. Table S2. Structures of all tested molecules (note that NQN, which has not been tested, is also shown). Table S3. *K*_i_ values for CZP and OZP at different receptors and hM4D(Gi) EC_50_ (bold and indicated with an arrow).
