# Supplementary material for: Olanzapine: A potent agonist at the hM4D(Gi) DREADD amenable to clinical translation of chemogenetics
Source: Sci Adv. 2019 Apr 17;5(4):eaaw1567. doi: 10.1126/sciadv.aaw1567 (PMC6469940; doi:10.1126/sciadv.aaw1567)
Supplement: http://advances.sciencemag.org/cgi/content/full/5/4/eaaw1567/DC1 [file supp_5_4_eaaw1567__index.html]

Science Advances | Science Advances

## Supplementary Materials

**This PDF file includes:**

- Fig. S1. Docking poses of OZP and CPX.
- Table S1. Hit list of the 3D- and 2D-based screens.
- Table S2. Structures of all tested molecules (note that NQN, which has not been tested, is also shown).
- Table S3. *K*i values for CZP and OZP at different receptors and hM4D(Gi) EC50 (bold and indicated with an arrow).

Download PDF

**Files in this Data Supplement:**

- Adobe PDF - aaw1567\_SM.pdf
