# Supplementary material for: Olanzapine: A potent agonist at the hM4D(Gi) DREADD amenable to clinical translation of chemogenetics
Source: Sci Adv. 2019 Apr 17;5(4):eaaw1567. doi: 10.1126/sciadv.aaw1567 (PMC6469940; doi:10.1126/sciadv.aaw1567)

Science Advances
AAAS

advances.sciencemag.org/cgi/content/full/5/4/eaaw1567/DC1

Supplementary Materials for

Olanzapine: A potent agonist at the hM4D(Gi) DREADD amenable to clinical translation of chemogenetics

Mikail Weston, Teresa Kaserer, Angela Wu, Alexandre Mouravlev, Jenna C. Carpenter, Albert Snowball, Samuel Knauss, Melanie von Schimmelmann, Matthew J. During, Gabriele Lignani, Stephanie Schorge, Deborah Young, Dimitri M. Kullmann*, Andreas Lieb*

*Corresponding author. Email: d.kullmann@ucl.ac.uk (D.M.K.); a.lieb@ucl.ac.uk (A.L.)

Published 17 April 2019, *Sci. Adv.* **5**, eaaw1567 (2019)
DOI: 10.1126/sciadv.aaw1567

This PDF file includes:

Fig. S1. Docking poses of OZP and CPX.
Table S1. Hit list of the 3D- and 2D-based screens.
Table S2. Structures of all tested molecules (note that NQN, which has not been tested, is also shown).
Table S3. K_i values for CZP and OZP at different receptors and hM4D(Gi) EC_{50} (bold and indicated with an arrow).

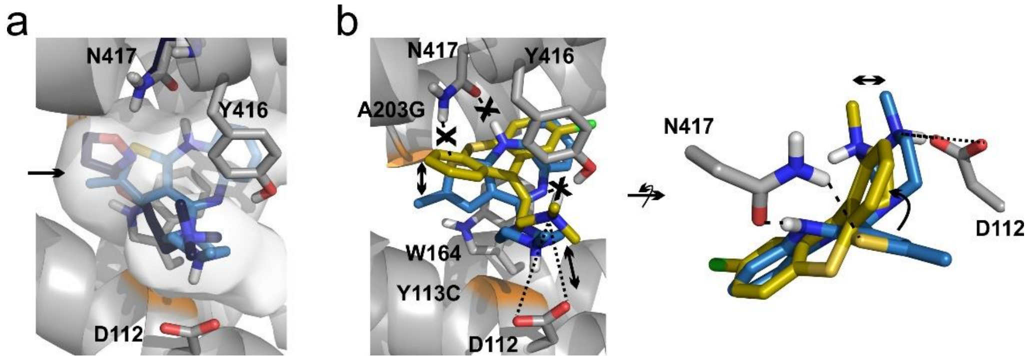

Fig. S1. Docking poses of OZP and CPX.
a) The methyl-group of OZP (blue sticks) extends into a side pocket (white surface, highlighted by an arrow) occupied by iperoxo (black sticks, copied from the crystal structure in complex with hM2 (PDB entry 4MQS (25)). **b)** The docking pose of CPX (yellow sticks) suggests that none of the hydrogen bonds observed for OZP (blue sticks) can be formed. The basic moiety of CPX is positioned further away from D112 and the two compounds adopt a different 3D configuration. The model of the active hM4D(Gi) is shown as gray cartoon and sticks and the Y113C and A203G mutations are highlighted in orange.

Table S1. Hit list of the 3D- and 2D-based screens. Only FDA/EMA-approved molecules are listed (applicable for CHEMBL databank). The drug name, Tanimoto Combo (for 3D) or similarity (for 2D), as well as the screen database, and the individual identifiers (ID) are indicated. (Drugbank 5.0.7 (38), CHEMBL (23), accessed 21.June 2017)

	rank	drug name	TanimotoCombo / similarity	database	ID
3D	1	Olanzapine	1.47	drugbank	DB00334
3D	2	Promazine	1.383	drugbank	DB00420
3D	3	Chlorpromazine	1.374	drugbank	DB00477
3D	4	Clozapine	1.363	drugbank	DB00363
3D	5	Tripelennamine	1.338	drugbank	DB00792
3D	6	Diphenhydramine	1.33	drugbank	DB01075
3D	7	Chloropyramine	1.32	drugbank	DB08800
3D	8	Alimemazine	1.312	drugbank	DB01246
3D	9	Chlorprothixene	1.305	drugbank	DB01239
3D	10	Orphenadrine	1.296	drugbank	DB01173
2D	1	Clozapine	88.9	CHEMBL	CHEMBL42
2D	2	Amoxapine	84.52	CHEMBL	CHEMBL1113
2D	3	Perlapine	80.34	CHEMBL	CHEMBL340801
2D	4	Loxapine	77.78	CHEMBL	CHEMBL831
2D	5	Fluperlapine	77.48	CHEMBL	CHEMBL63756
2D	6	Quetiapine	74.52	CHEMBL	CHEMBL716
2D	7	Metiapine	73.93	CHEMBL	CHEMBL2106892
2D	8	Clothiapine	72.85	CHEMBL	CHEMBL304902

Table S2. Structures of all tested molecules (note that NQN, which has not been tested, is also shown).

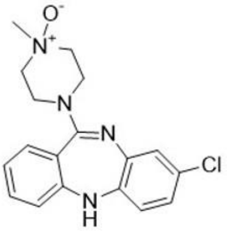

Clozapine-N-oxide

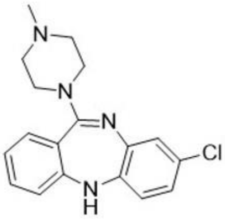

Clozapine

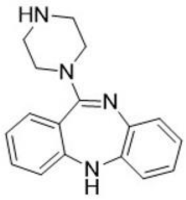

Compound 21

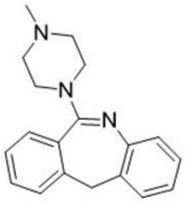

Perlapine

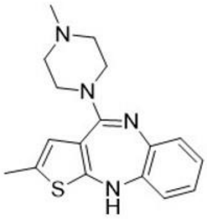

Olanzapine

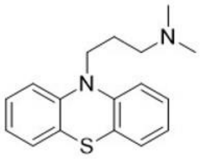

Promazine

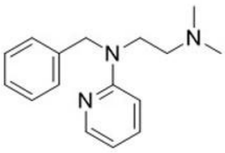

Tritilennamin

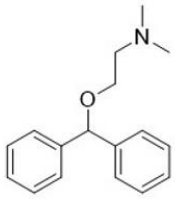

Diphenhydramine

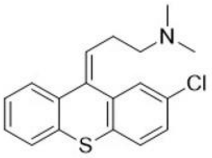

Chlorprothixen

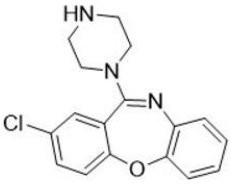

Amoxapine

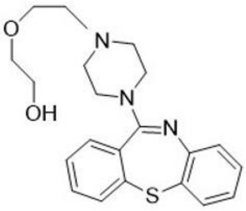

Quetiapine

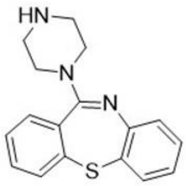

Norquetiapine

Table S3. K_i values for CZP and OZP at different receptors and hM4D(Gi) EC_{50} (bold and indicated with an arrow). Data from the PDSP K_i Database (https://pdsp.unc.edu/databases/kidb.php; access date 8th February 2019). NA: not available.

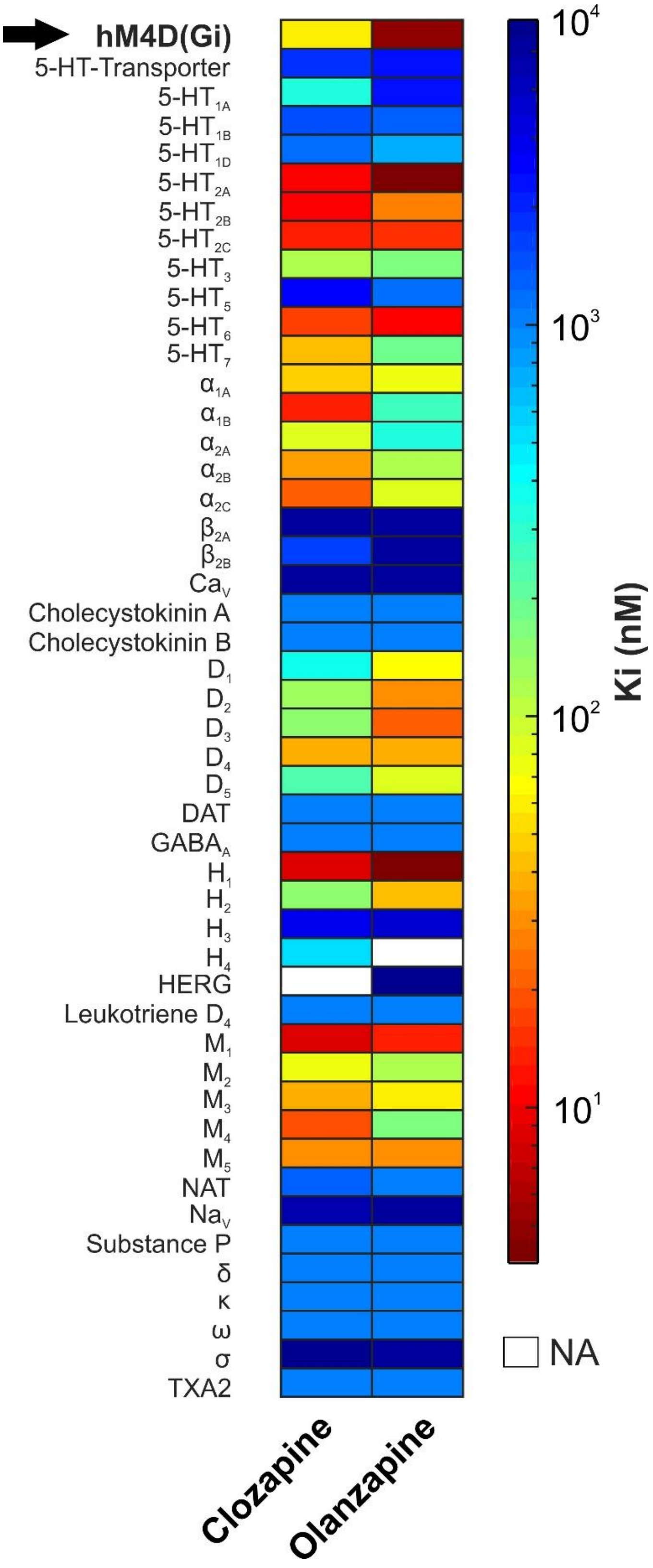

Supplement: Download PDF [file aaw1567_SM.pdf]
